# Preparation of Edible Colorant Lake Using Calcium Carbonate and β-Carotene: Structural Characterization and Formation Mechanism Study

**DOI:** 10.3390/foods13071050

**Published:** 2024-03-29

**Authors:** Yuhan Liu, Le Jing, Jiaqi Cui, Dongdong Yuan, Chengtao Wang

**Affiliations:** 1 Beijing Engineering and Technology Research Center of Food Additives, School of Food and Health, Beijing Technology and Business University, Beijing 100048, China; liuyuhan_210@163.com (Y.L.); 2130022077@st.btbu.edu.cn (L.J.); c18647362147@163.com (J.C.); wctbtbu@hotmail.com (C.W.); 2 Key Laboratory of Geriatric Nutrition and Health, Ministry of Education, School of Food and Health, Beijing Technology and Business University, Beijing 100048, China; 3 China Food Flavor and Nutrition Health Innovation Center, School of Food and Health, Beijing Technology and Business University, Beijing 100048, China

**Keywords:** β-carotene, colorant lake, calcium carbonate, kinetic adsorption, isothermal adsorption, physical adsorption

## Abstract

This study prepared a novel β-carotene colorant lake using calcium carbonate (CaCO_3_) and investigated the lake formation process and its basic characteristics. Kinetic adsorption analysis confirmed that medium pH (9.3) and medium temperature (40 °C) were more suitable for lake preparation, while desorption occurred, possibly due to crystalline transformation of CaCO_3_. The isothermal analysis and model fitting results suggested that the β-carotene and CaCO_3_ particles combined via a monolayer adsorption process driven by physical force. Electrostatic attraction likely participated in the process due to the net negative surface charges of β-carotene dispersion and positively charged groups on the CaCO_3_ particle surfaces. Ethanol, ultrasonic treatment, and drying method significantly influenced the immobilization efficiency (IE) of β-carotene in the lake and light stability of the lake, without affecting its crystal form. The thermogravimetric analysis (TGA) and differential scanning calorimetry (DSC) curves confirmed absorption of β-carotene onto CaCO_3_. Fourier-transform infrared spectroscopy (FTIR) and X-ray photoelectron spectroscopy (XPS) analyses indicated no obvious chemical bonds between β-carotene and CaCO_3_. Energy-dispersive spectroscopy (EDS) confirmed the presence of β-carotene on surfaces but not in the interior of the CaCO_3_ particles. The adsorption of β-carotene by calcium carbonate was further confirmed to be a physical adsorption on surface.

## 1. Introduction

The application of natural ingredients in food production is gaining increasing attention recently due to their superior safety, nutritional functionalities, and biological properties compared to synthetic materials [1,2,3]. As a typical example, β-carotene is favored as a pigment for food use due to its versatile biological functionalities including antitumor, antiaging, antioxidative, free radical scavenging properties, improving immunity, promoting embryo development, and maintaining vision [4]. β-carotene is an oil-soluble pigment, which covers the orange to red chroma depending on its concentration. It is widely used in oily food products, such as butter, margarine, refined fish pulp products, and instant noodles. β-carotene is composed of 11 conjugated double bonds and two ionone rings at both ends. Its structure is extremely unstable, and it is prone to degradation and isomerization in the presence of light, oxygen, free radicals, high temperatures, and metal ions [5]. Encapsulation technologies, including emulsion [6], β-cyclodextrin [7], microcapsules [8], liposomes [9], and solid lipid nanoparticles [10], have been extensively employed to encapsulate β-carotene. These methods aim to coat β-carotene to the maximum extent to achieve the most efficient protection and successively increase its biological activity, which is widely achieved in the literature. Zehra et al. investigated the inclusion of β-carotene by cyclodextrins, which protected β-carotene from UV-mediated oxidation and ensured its biological activity [11]. Alma et al. prepared β-carotene-chitooligosaccharides complexes by mechanochemical methods, and the complexation did not affect the free radical scavenging activity of β-carotene and ensured the water solubility of β-carotene [12]. However, the forementioned encapsulation technologies and their aims are inconsistent with the dyeing purpose that requires adequate β-carotene exposure. Lake pigment technology is more suitable to protect colorants and their dyeing ability. However, to date, only water-soluble pigments have been transformed into colorant lakes in the literature, necessitating the exploration of efficient technology to prepare colorant lakes for oil-soluble pigments, such as β-carotene.

Aluminum hydroxide is the traditional, most common adsorbent for food-grade colorant lake preparation [13]. However, in recent years, several studies have shown that excessive aluminum accumulation in the body causes aluminum poisoning and aggregation in the brain, liver, spleen, kidneys, thyroid, and other tissues and organs. Only 10–15% of consumed aluminum is excreted. The remainder accumulates in the body and interacts with proteins, enzymes, and other components to affect various biochemical reactions [14]. Aluminum deposition in brain and nerve cells causes memory loss, mental decline, and slow reactions, possibly accelerating the aging process and inducing neurodegenerative diseases, such as Alzheimer’s disease [15], Parkinson’s disease [16], dialysis encephalopathy, and amyotrophic lateral sclerosis [17]. Aluminum deposition in bones can lead to osteoporosis, fractures, and joint pain, while deposition in skin can reduce skin elasticity and increase wrinkles [18,19,20]. Therefore, the current food additive standard of China, <Standard for the Use of Food Additives> (GB 2760-2014) [21], has reduced the application range and abolished some aluminum additives, compared with the previous version (GB 2760-2011) [22,23]. For example, the application of indigo carmine aluminum hydroxide lake has been restricted. Therefore, our previous study developed a calcium-carbonate-based colorant lake as a substitute for the aluminum-hydroxide-based lake [24]. CaCO_3_ presents no health risk and offers additional benefits as a calcium supplement. Previous studies have used CaCO_3_ as an adsorbent due to its multiple surface modifiability and significant surface charge [25,26,27]. Studies on indigo carmine have shown that coordination bond formation between sulfonic acid groups and calcium ions can form Ca^2+^-IC complexes [28]. Subsequent analysis of CaCO_3_ and *Monascus* red pigments (MPs) binding indicated physical attraction as the main driving force behind MPs and CaCO_3_ complex formation, most likely the electrostatic attraction between Ca^2+^ and glutamate residues in MPs. MPs-CaCO_3_ complex formation is a multistep process involving adsorption, aggregation, crystallization, and desorption [24].

A preliminary experiment in our laboratory confirmed that coprecipitation could be used to form an ideal β-carotene-CaCO_3_ colorant lake. Although aluminum hydroxide could precipitate with β-carotene, the color intensity and yield of the precipitation were relatively low, making it unsuitable for producing colorant lakes for oil-soluble pigments. The performance variation of the two substrates when constructing lakes for oil-soluble pigments could be due to their microstructural disparity, which will be further explained in future studies.

This study successfully prepared a β-carotene-CaCO_3_ colorant lake for the first time, which was characterized in terms of light stability, IE, and morphological properties. The influence of ethanol, ultrasonic treatment, and drying method on the colorant lake were investigated. Additionally, the kinetic and isotherm adsorption curves were examined to assess the CaCO_3_ and β-carotene interaction in the reaction solution, while the existing states of the β-carotene in the lake were analyzed via ultraviolet/visible spectroscopy (UV/Vis), Fourier-transform infrared spectroscopy (FTIR), thermogravimetric analysis (TGA), differential scanning calorimetry (DSC), X-ray photoelectron spectroscopy (XPS), and energy-dispersive spectroscopy (EDS).

## 2. Materials and Methods

### 2.1. Subsection

Natural β-carotene (96% *w*/*w*) and chemicals of analytical grade, including calcium chloride (96% *w*/*w*), sodium carbonate (99.5% *w*/*w*), sodium hydroxide (99% *w*/*w*), and n-hexane (97% *w*/*w*), were obtained from Macklin Inc. (Shanghai, China). The ethanol (99.7% *w*/*w*) and hydrochloric acid (37% *w*/*w*) were supplied by Beijing Chemical Plant (Beijing, China). A laboratory water purification system (HYP-QX-UP, Huiyipu Ltd., Beijing, China) was employed to produce the deionized (DI) water used for the solutions required for the experiment.

### 2.2. Preparation of the β-Carotene-CaCO_3_ Colorant Lake

The β-carotene-CaCO_3_ colorant lakes were prepared via a suspension–precipitation method. Here, 2.0 g of sodium carbonate was dissolved in a 198.0 g water/ethanol solution, while 2.1 g of calcium chloride was dissolved in a 25.0 g water/ethanol solution. The pH of the solutions was adjusted to 9.3 using 1 mol/L HCl and NaOH solutions. The two solutions were heated and kept in a water bath at 40 °C before use. Next, 50.0 mg of β-carotene was added to the sodium carbonate solution, followed by shearing at 18,000 r/min for 90 s (IKA ULTRA-TURRA ^®^ T25 digital, Staufen, Germany) to form a suspension solution with a β-carotene. Then, the calcium chloride solution was mixed with the suspension solution while shearing at 18,000 r/min for 150 s. Since the high-speed shearing inevitably increased the solution temperature, an ice-water bath was employed to control the temperature below 45 °C. After shearing, the reaction solutions were centrifuged at 5000 r/min for 15 min to obtain the CaCO_3_ precipitates adsorbed with pigments. The sediment was dried in a vacuum drying oven at 45 °C for 12 h and ground in a mortar to acquire an edible β-carotene-CaCO_3_ colorant lake.

### 2.3. Quantification of the β-Carotene in the Lake

Here, 50.0 mg of the colorant lake was dispersed into a 1 mL acid solution with a hydrochloric acid concentration of 1 mol/L for complete dissolution. Then, 3 mL of an organic solvent (1 mL of ethanol + 2 mL n-hexane) was added and mixed, followed by shaking using a vortex shaker for 45 s to dissolve the β-carotene into the organic phase. The mixture was left to stand to allow complete phase separation, after which the upper layer (solvent phase) was collected. The water phase (lower phase) was extracted by repeating this process twice more, after which the solvent phases of the three extractions were combined and filtered through 0.22 μm organic membranes. The absorbance of the filtered solution was measured at 450 nm using a spectrophotometer (Shimadzu UV3600Plus, Kyoto, Japan). High-purity β-carotene (99% *w*/*w*) was used to establish the standard linear curve of the β-carotene at 450 nm absorbance. Then, the amount of β-carotene immobilized by calcium carbonate was calculated and denoted as M_1_ (mg), and the immobilization efficiency (IE) of β-carotene (mg/g) was calculated using Equation (1):

(1) $$\mathrm{IE}=M_{1}/M_{2}\times 100\%$$

where M_1_ is the mass of the β-carotene detected in the lake (mg), and M_2_ is the mass of the lake, which is 0.05 g.

### 2.4. Kinetic Adsorption Analysis

(1) The influence of temperature: The suspension and calcium chloride solutions were prepared as described in Section 2.2. The solutions were preheated in a water bath at 30 °C before mixing and shearing at 18,000 r/min for 90 s. Next, a magnetic stirrer with a temperature-control function was used to stir the reaction solutions at 30 °C. At predetermined time points, the reaction solution was centrifuged, as described in Section 2.2, to obtain the sediment. The experiment lasted 48 h (2880 min). The sediment collected at each time point was dried in a vacuum drying oven at 45 °C for 12 h and ground in a mortar to obtain the CaCO_3_ lake. The quantity of β-carotene in the lake (M_1_) was measured using the method described in Section 2.3, while the adsorbed β-carotene at each time point (q_t_) was calculated using Equation (2). The q_t_ was plotted against time to obtain the adsorption kinetic curves, which were also determined at 40 °C and 50 °C using the same method employed for the 30 °C measurements but with modifications regarding the water bath temperature and stirring parameters.

(2) $$q_{t}=M_{1}/M_{c}\times 100\%$$

where M_1_ is the mass of the β-carotene detected in the lake (mg), and M_c_ is the mass of the CaCO_3_ in the lake (g). 

(2) The influence of pH: The kinetic curves were determined using the same method described above, with modifications regarding the temperature, which was set at a constant 40 °C, while pH values of 8, 9.3, and 10 were used to investigate the influence of pH on the kinetic curve of lake formation.

### 2.5. Isothermal Adsorption Analysis

To prepare the working solutions, 0.80 g of Na_2_CO_3_ was dissolved in 79.2 g of DI water. NaOH/HCl was used to adjust the solution pH to 9.3, after which 0.84 g of CaCl_2_ was dissolved in 10.0 g of DI water to prepare the CaCl_2_ for later use. The method described in Section 2.2 was used to prepare a series of lakes by adding different concentrations of β-carotene, i.e., 0.5 mg/mL, 1 mg/mL, 1.5 mg/mL, 2 mg/mL, 3 mg/mL, 4 mg/mL, 5 mg/mL, 6 mg/mL, 7 mg/mL, 8 mg/mL, 9 mg/mL, and 10 mg/mL. The β-carotene content in the lakes was measured using the method described in Section 2.3, while adsorbed β-carotene quantity per gram of CaCO_3_ at equilibrium (q_e_ (mg/g)) was calculated using Equation (2). The q_e_ is actually the qt at equilibrium stage. The unabsorbed β-carotene concentration in the reaction solution (C_e_ (mg/mL)) was calculated using Equation (3). The isothermal adsorption curve was drawn by plotting q_e_ against C_e_:

(3) $$C_{e}=\frac{C_{1}V-q_{e}\times m_{C{\mathrm{aC}O}_{3}}}{V}$$

where C_1_ is the concentration of β-carotene added to the solution, C_e_ is the concentration of unabsorbed β-carotene at equilibrium (mg/mL), $m_{{CaCO}_{3}}$ is the amount of calcium carbonate produced (g), and V is the solution volume (mL).

To clarify the adsorption process between the CaCO_3_ and β-carotene, the isothermal adsorption data were fitted into Langmuir and Freundlich isothermal adsorption curve equations, as shown in Equations (4)–(6):

(4) $$\mathrm{Langmuir}:\;\frac{C_{e}}{q_{e}}=\frac{1}{q_{\max}\times K_{L}}+\frac{C_{e}}{q_{\max}}$$

(5) $$R_{L}=\frac{1}{1+{(K}_{L}\times C_{0})}$$

(6) $$\mathrm{Freundlich}:\;\ln(q_{e})=\ln(K_{f})+\frac{\ln(C_{e})}{n}$$

where q_max_ is the maximum value of q_e_ (mg/g), K_L_ is the adsorption equilibrium constant of the Langmuir model (L/mg), n is the Freundlich constant, representing adsorption strength, K_f_ is the adsorption equilibrium constant of the Freundlich model (mg^1−1/n^·g^−1^·L^1/n^), and C_0_ is the initial β-carotene concentration in solution after each addition (mg/L).

### 2.6. Preparation of Lake with Varying Ethanol Contents, Ultrasonic Treatments, and Drying Methods

(1) Varying ethanol content: The lake was prepared using the method described in Section 2.2. The ethanol volume ratio in the water/ethanol solution were set to 0% (water: ethanol = 1:0), 10% (water: ethanol = 9:1), 20% (water: ethanol = 4:1), 30% (water: ethanol = 7:3), 40% (water: ethanol = 3:2), and 50% (water: ethanol = 1:1), respectively.

(2) Varying ultrasonic treatments: The lake was prepared using the method as described in Section 2.2, which was modified by adding ultrasonic treatment (Kunshan Shumei KQ2200DE, Kunshan, China) after the second shearing operation at 2 min, 4 min, 6 min, 8 min, and 10 min, respectively.

(3) Varying drying methods: The lake was prepared using the method described in Section 2.2, which was modified by employing freeze drying, vacuum drying, and oven drying, respectively, to dry the sediment.

### 2.7. Determination of the Light Stability

The lake sample was placed in a light incubator (Shanghai shbo-xun SPX-150B-Z, Shanghai, China) with stable, simulated natural light illumination at 12,000 LX and a temperature of 25 °C. The CIE L*a*b* color scale was used for evaluation. A color difference meter (Konica Minolta CM-3610A, Tokyo, Japan) was used to determine the change in redness (Δa*) and total color difference (ΔE) after 48 h. Lower Δa* and ΔE absolute values indicated higher color stability.

### 2.8. Microscopic Morphology Analysis

The microscopic morphology of the β-carotene-CaCO_3_ colorant lake was observed using SEM. The lake was dried in an oven at 45 °C for 12 h to minimize its moisture content before observation. The dried powder samples were sputtered with gold, and their microscopic morphology was observed using a microscope (Hitachi S-4800, Tokyo, Japan). The prepared lake was placed in a mortar, and liquid nitrogen was poured into the mortar to freeze the lake sample, which was then quickly ground. The C, Ca, and O distribution on the surfaces and cross-sections of the complex lake particles was analyzed via EDS using point-scanning mode.

### 2.9. Crystal Structure Analysis

The crystal morphology of the CaCO_3_ lake was analyzed via X-ray diffraction. The sample was scanned at a speed of 2°/min in a range of 5 to 90° (2θ) using the fine scanning mode of a diffractometer (Rigaku SmartLab SE, Tokyo, Japan). The scan data were analyzed using MDI Jade 6.

### 2.10. Zeta Potential Measurement

The lake was dispersed in DI water, after which about 1 mL of the solution was injected into a capillary zeta potential cell using a syringe. The cell was placed in a Malvern Zetasizer Nano ZS90 potentiometer (Malvern Instruments, Malvern, UK) to determine its surface charge, while the potential was measured at ambient temperature. The zeta potential of the β-carotene particles was immediately determined after the shearing operation to disperse β-carotene into the sodium carbonate solution, as shown in Section 2.2.

### 2.11. Particle Size Measurement

A Malvern Mastersizer 2000 laser particle size analyzer (Malvern Instruments, Malvern, UK) was used to determine the particle sizes at 25 °C, and the volume-weighted average diameter, d_4,3_, was recorded.

### 2.12. BET Surface Area Measurement

This process was performed using a BET machine (Quanta Autosorb-iQ, Houston, TX, USA). Before the measurement, the lake samples were ground to obtain smaller particles. The lake and CaCO_3_ powders were degassed at 80 °C, and the N_2_ adsorption data were determined and analyzed.

### 2.13. TGA and DSC Analyses

A synchronous thermal analyzer (TA Instruments Q600, New Castle, DE, USA) was used for the TGA and DSC analyses. The lake samples were placed on a standard aluminum pan and sealed tightly with a perforated aluminum sheet. The samples were heated from room temperature to 800 °C at a constant rate of 10 °C/min using nitrogen as a gas atmosphere. The weight change and heat flow signal were recorded and analyzed [29].

### 2.14. FTIR Analysis

The β-carotene-CaCO_3_ colorant lake was dried in a vacuum drying oven at 45 °C for 12 h before measurement. The sample was mixed with potassium bromide at a mass ratio of 1:150 and pressed into pellets for analysis. The light transmittance was determined in a range of 400 cm^−1^ to 4000 cm^−1^ at a resolution of 2 cm^−1^ using a Nicolet iS5 FTIR spectrometer (Thermo Fisher, Waltham, MA, USA).

### 2.15. XPS Analysis

The prepared colorant lake was dried in a vacuum drying oven at 45 °C for 12 h before measurement. XPS analysis was performed using an Escalab 250Xi+ X-ray photoelectron spectrometer (Thermo Fisher, Waltham, MA, USA), and C, O, and Ca levels were analyzed.

### 2.16. Statistical Analysis

The data were processed and graphed using Origin 2021. All assays were performed in triplicate, and the results are presented as mean ± standard deviation (SD). Significance analysis was performed using IBM SPSS Statistics 26, while differences were regarded as significant when *p* < 0.05.

## 3. Results and Discussion

### 3.1. Kinetic Adsorption Curve

Since β-carotene is suspended as small particles in aqueous solutions rather than being fully dissolved, the process of combining it with CaCO_3_ does not involve typical adsorption. Assuming that a β-carotene suspension particle was a whole entity involved in the adsorption process, standard adsorption evaluation methods could be employed, such as kinetic and isothermal curve analyses. 

**Figure 1:**
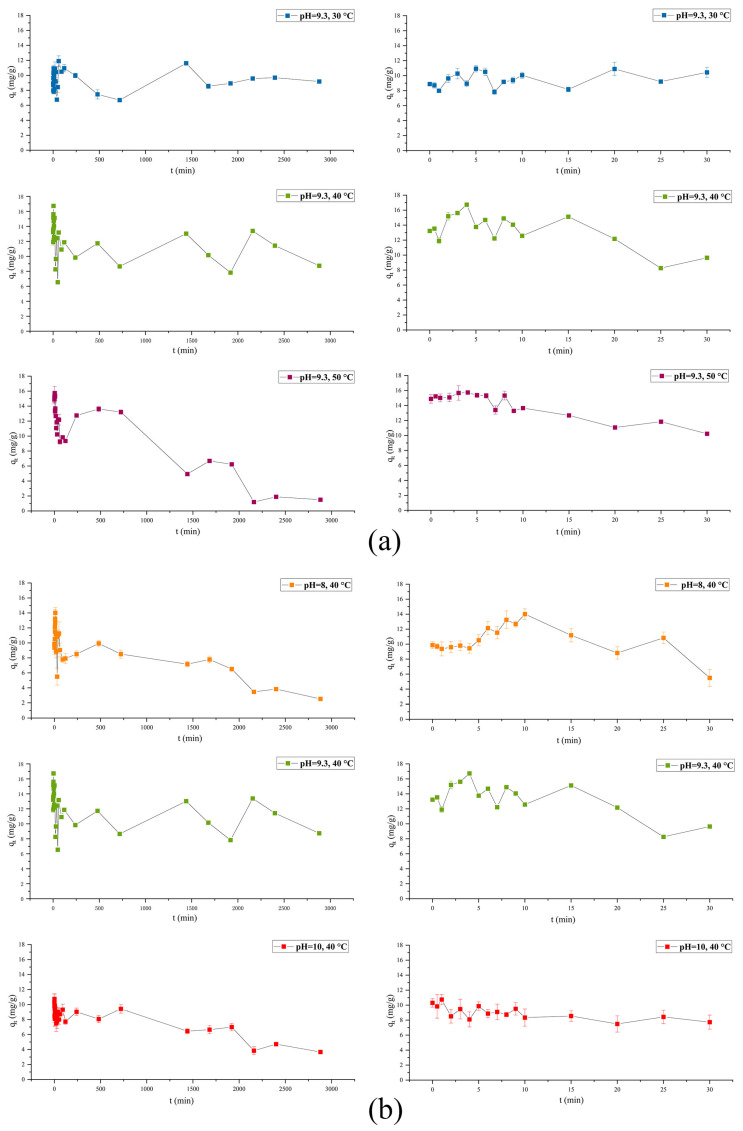
The influence of temperature (**a**) and pH (**b**) on the kinetic adsorption curve (left: 0–2880 min, right: 0–30 min).

Figure 1a,b illustrate the kinetic curves. The temperature variation from 30 °C to 50 °C distinctly influenced the kinetic curves (Figure 1a). Minimal q_t_ variation was evident at 30 °C, from about 7.827 mg/g to 10.928 mg/g in the first 30 min, indicating stable β-carotene and CaCO_3_ binding. From 30 min to 2880 min, the q_t_ fluctuation range increased from 6.696 mg/g to 11.899 mg/g. At 40 °C, the q_t_ displayed an initial increase to a maximum value of 16.731 mg/g at 5 min, followed by a slight decrease over 30 min, after which the q_t_ fluctuated in a range of 6.545 mg/g to 13.388 mg/g. At 50 °C, the q_t_ remained almost constant during the first 6 min at about 15 mg/g, then gradually decreased to around 10 mg/g at 30 min and approximately 2 mg/g at the end of the experiment. The q_t_ values of all the curve peaked during the first 10 min of the adsorption reaction, after which they generally declined until the end of the experiment. This was especially evident for the curve at 50 °C. This was consistent with reports on an MPs-CaCO_3_ lake [24]. The q_t_ decrease indicated β-carotene desorption from the CaCO_3_ particles, which was related to the crystalline CaCO_3_ transformation from an amorphous form to calcite. In summary, a medium temperature (40 °C) increased the β-carotene content in the lake compared to a lower temperature (30 °C) and facilitated higher adsorption stability (less desorption) compared to a higher temperature (50 °C). Therefore, 40 °C was selected for lake preparation in the subsequent experiment. 

Figure 1b shows the influence of pH on the kinetic curves. The pH can affect the functional groups, charge level, and ion form of the adsorbent, consequently impacting its adsorption capacity [30]. At pH 8 and 9.3, the q_t_ exhibited an initial increase, reaching the maximum levels, followed by a decline. However, at pH 10, the maximum level appeared at the beginning of the measurement, after which the q_t_ decreased throughout the remainder of the test. The maximum qt values at pH 8, 9.3, and 10 were 14.008 mg/g, 16.731 mg/g, and 10.745 mg/g, respectively, indicating that pH 9.3 was more suitable for lake preparation. A previous study involving the preparation of an MPs-CaCO_3_ lake also investigated the pH of the reaction solution, confirming that pH 10.5 was optimal due to the highest q_t_ level [24]. The pH values of achieving maximum of q_t_ for MPs and β-carotene are different but close. The difference reflects the disparity between the two pigments in terms of chemical composition, and the proximity of the pH values may suggest similar driving forces or manners existing in the adsorption processes of the two pigments onto CaCO_3_. Since the *Monascus* pigments were negatively charged due to their carboxyl groups in a basic pH range, they could be absorbed by CaCO_3_. The CaCO_3_ surface charge is determined by the presence of -Ca^+^ and -CO_3_^−^ sites on its surface and their possible hydrolysates, such as -CaOH, -Ca (OH_2_)^+^, -CO_3_H, and -CO_3_ (OH_2_)^−^ [31]. The zeta potentials of β-carotene suspension, calcium carbonate particles, and lake particles (β-carotene-CaCO_3_ complex particles) with different pH values to those in the preparation process are shown in Table 1. It is evident that all of the three particles exhibit negative charges on surfaces. The zeta potential of calcium carbonate was higher than that of the color lake and β-carotene suspension. The results indicated that the presence of -Ca^+^ site on the surface of calcium carbonate and its possible cationic hydrolysate adsorbed pigment suspensions that were negatively charged, resulting in more net negative charges on the surface of the complex lake particles [24,32]. The results verified the important role of electrostatic attraction as a driving force for the formation of β-carotene-CaCO_3_ complex particles.

### 3.2. Isothermal Curve of Adsorption

**Figure 2:**
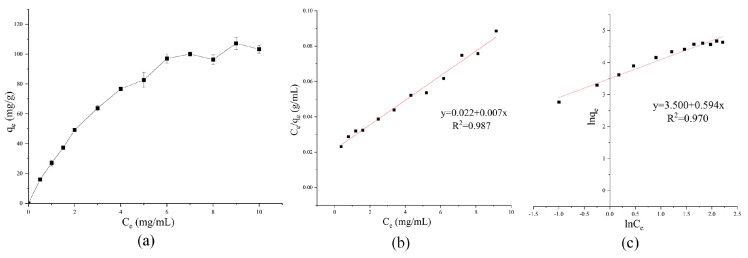
The isothermal adsorption curves of the β-carotene-CaCO_3_ colorant lake (40 °C) (**a**), linear Langmuir equation data fitting (**b**), and linear Freundlich equation data fitting (**c**).

Both of the q_e_ and C_e_ values increased after adding β-carotene to the reaction solution (Figure 2a). The q_e_ increased rapidly and exhibited a steep slope as the C_e_ rose from 0 mg/mL to 6 mg/mL. At levels higher than 6 mg/mL, the slope flattened abruptly and leveled off. When the added β-carotene quantity exceeded 7 mg/mL, the CaCO_3_ particles were saturated, and the q_e_ reached a maximum value of about 100.02 mg/g, indicating that every gram of CaCO_3_ could immobilize 100.20 mg of β-carotene. 

Figure 2b,c show the fitting of isothermal data into the linear Langmuir and Freundlich equations. The R^2^ values indicated that the data fitted both of the Langmuir and Freundlich models well. However, the Langmuir model displayed slightly better correlations. The q_max_, K_L_, and R_L_ values of the Langmuir model were calculated as 142.86 mg/g, 0.32 mL/mg, and 0.24~0.86, respectively, using Equations (4) and (5). According to the Langmuir adsorption theory, the adsorption is a monolayer adsorption process, and when the R_L_ value is less than 1, the adsorption process is favorable [33]. For the Freundlich model, K_f_ was calculated to be 33.12, and n was calculated to be 1.68. According to the Freundlich adsorption theory, when n > 1, adsorption is generally a physical process [24,34]. The results suggest that the adsorption between the β-carotene droplets and CaCO_3_ is likely a monolayered process driven by physical molecular forces. The conclusion was partially consistent with the isothermal MPs-CaCO_3_ absorption analysis, which agreed with the Freundlich model but did not fit into the Langmuir model [24]. Compared with the dissolved *Monascus* pigment molecules, the suspended β-carotene particles were not expected to form multiple adsorption layers on the CaCO_3_ particle surfaces due to their significant size.

### 3.3. Influence of Ethanol on the β-Carotene-CaCO_3_ Lake Preparation

**Figure 3:**
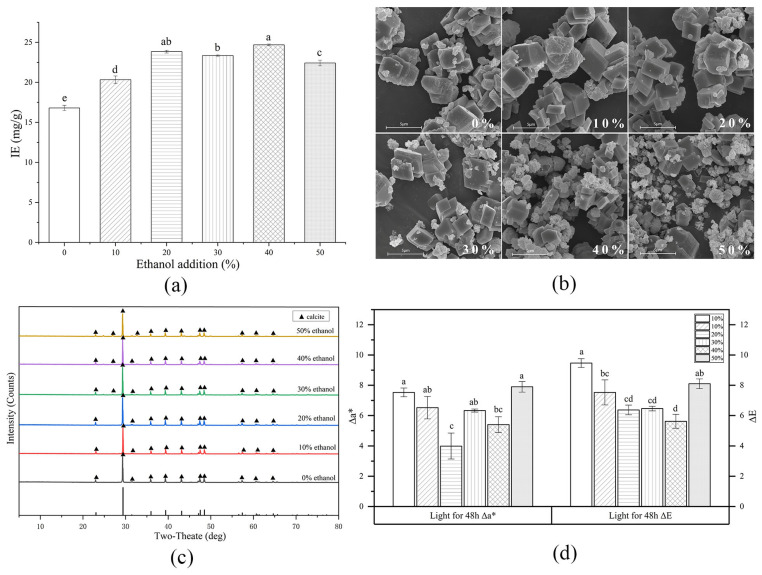
The influence of ethanol on the IE (**a**), micromorphology (**b**), crystalline form (**c**), and light stability (**d**) of the β-carotene-CaCO_3_ lake. (Values not sharing the same superscript letters in (**a**) and (**d**) are significantly different (*p* < 0.05).)

Studies have shown that the appearance of ethanol significantly influences CaCO_3_ formation and crystalline transformation [35,36]. Therefore, it is necessary to clarify the effect of ethanol on the properties of CaCO_3_-based colorant lake. Figure 3a shows the impact of the ethanol concentration in the lake-forming solution on the β-carotene IE. Due to the poor solubility of Na_2_CO_3_ and CaCl_2_ in solutions containing over 50% ethanol, only lower ethanol concentrations were examined. Ethanol addition significantly enhanced the β-carotene IE, reaching maximum levels after adding 20% ethanol, while the IE fluctuated at higher ethanol concentrations. Small and uniform size could facilitate the adsorption of particles [37], while the introduction of low-concentration ethanol resulted in the reduction in pigment particle size and the enhancement of absorbability (Figure 3a). Figure 3b shows the effect of the ethanol concentration on the micromorphology of the lake. The CaCO_3_ in the lake displayed a clear outline with flat and smooth surface, exhibiting typical calcite topography as reported in the literature [38]. The XRD analysis (Figure 3c) confirmed that only one type of crystal form of CaCO_3_, calcite, was present in the lake, regardless of the ethanol concentration. In addition to calcite, the SEM image showed some particles that appeared irregular in shape but displayed a wide range of sizes, which were likely immobilized β-carotene particles. The β-carotene dispersed on the CaCO_3_ particle surfaces in both the ethanol-added and ethanol-free lakes. As the ethanol content increased, a decrease in particle size and enhanced dispersion of particles were observed (Figure 3a). The findings showed that, even with the coprecipitation approach, there was a chance that the CaCO_3_ particles and β-carotene formed sediment due to various attraction forces rather than physical entanglement or direct physical entrapment, since a large amount of β-carotene appeared on the surface of CaCO_3_ particles. 

In light conditions, the Δa* value decreased as the ethanol concentration increased from 0% to 20%; however, beyond this threshold, the Δa* exhibited an increasing trend, with the minimum value evident at an ethanol concentration of 20%. Similarly, the ΔE initially decreased, followed by an increase at a higher added ethanol concentration. These findings demonstrated that lower ethanol concentrations enhanced the stability of the β-carotene-CaCO_3_ colorant lake, while higher concentrations detrimentally affected its stability.

### 3.4. Influence of Ultrasonic Treatment on the β-Carotene-CaCO_3_ Lake Preparation

**Figure 4:**
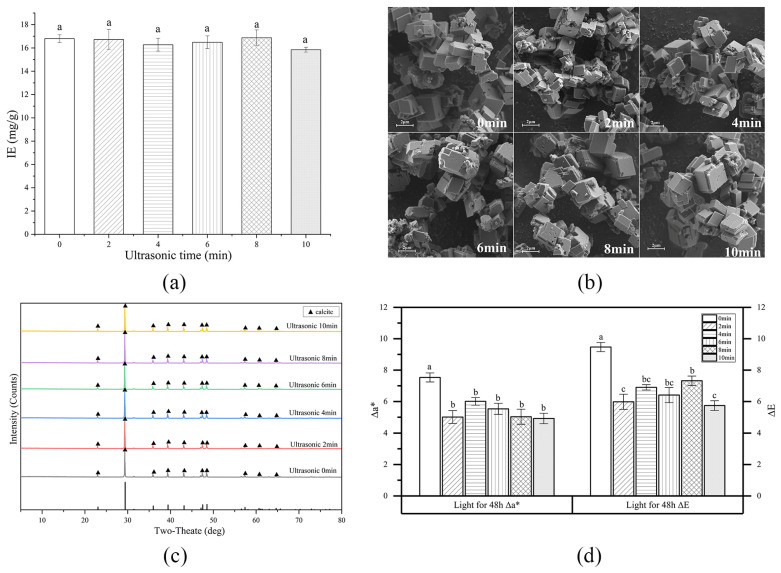
The influence of ultrasonic treatment on the IE (**a**), micromorphology (**b**), crystalline form (**c**), and light stability (**d**) of the β-carotene-CaCO_3_ lake. (Values not sharing the same superscript letters in (**a**) and (**d**) are significantly different (*p* < 0.05).)

It is worth investigating the effect of ultrasonic treatment on the β-carotene-CaCO_3_ lake properties since it can potentially improve CaCO_3_ particle uniformity [39]. Figure 4a shows the effect of ultrasonic treatment on the β-carotene IE. The lakes produced with varying ultrasonic treatment durations did not exhibit statistically significant IE variation (approximately 16.5 mg/g). This was significantly lower than the lakes prepared without ultrasonic treatment but with medium ethanol concentrations (Figure 3a) (*p* < 0.05). The CaCO_3_ particles in the lakes prepared via different ultrasonic treatments showed no distinct differences in size and morphology, and had similar size and morphology to those illustrated in Figure 3a, but exhibiting significantly fewer irregular particles, which was consistent with the lower β-carotene IE in the ultrasonically treated lake.

Figure 4d shows light stability of the β-carotene-CaCO_3_ lake prepared with different ultrasonic durations. The Δa* and ΔE values in the ultrasonic group after 48 h illumination were lower than those without ultrasonic treatment, showing that ultrasound significantly improved the colorant lake stability. Under illumination, longer ultrasonic treatment did not further improve the light stability. The results confirmed that ethanol addition was more efficient than ultrasonic treatment in improving the light stability since the former yielded the lowest Δa* value (about 4) among all the tests.

### 3.5. Influence of Drying Methods on β-Carotene-CaCO_3_ Lake Preparation

**Figure 5:**
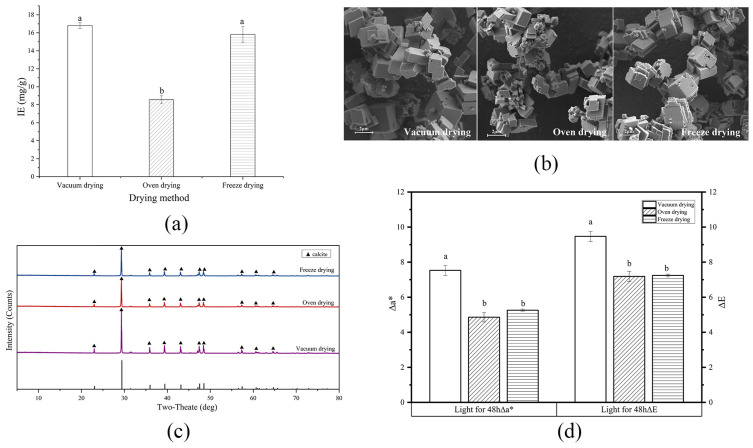
The influence of drying methods on the immobilization efficiency (IE) (**a**), micromorphology (**b**), crystalline form (**c**), and light stability (**d**) of the β-carotene-CaCO_3_ lake. (Values not sharing the same superscript letters in (**a**) and (**d**) are significantly different (*p* < 0.05).)

Figure 5a shows the IE of the β-carotene colorant lake prepared using different drying methods. The IE of the colorant lake prepared using vacuum drying was the highest (16.809 mg/g), followed by the sample prepared via freeze drying (15.819 mg/g), while that of the colorant lake prepared using oven drying was the lowest (8.562 mg/g). The results indicated that oven drying destroyed a large proportion of the immobilized β-carotene in the β-carotene-CaCO_3_ lake due to oxidation in the high-temperature, oxygen-rich environment. The IE discrepancy between vacuum and freeze drying was minimal (<1 mg/g) and not significant (*p* > 0.05). Figure 5b presents the micromorphology of the β-carotene-CaCO_3_ colorant lakes prepared in different drying conditions. The particles in the three lakes were similar, showing aggregation, size uniformity, and typical calcite topography. The XRD analysis confirmed that only calcite was present in the three kinds of lakes (Figure 5c). However, freeze drying demands higher energy input, more complicated operation, and a longer waiting time compared to vacuum drying. Therefore, vacuum drying was more suitable for colorant lake preparation.

Figure 5d shows the light stability of the lake products exposed to different drying methods. In light conditions, the Δa* and ΔE values of the sample prepared using vacuum drying were higher than samples prepared using oven drying and freeze drying, which had no significant difference between them (*p* > 0.0.5). However, the β-carotene IE of the colorant lake prepared with oven drying (8.562 mg/g) was about half that prepared with freeze drying (15.819 mg/g) and vacuum drying (16.809 mg/g). It stands to reason that, for the lake prepared by oven drying, its much lower IE contributed to its superiority in light stability. Thus, for all the drying methods, freeze drying is the best, considering its excellent performances in both IE and light stability. However, compared with freeze drying, vacuum drying has the characteristics of small equipment investment, large processing capacity, and simple operation [40]. For economical consideration, the vacuum drying, which only showed slightly insufficient light stability of prepared lake compared to the freeze drying method in the test, is also a good choice. 

In summary, ethanol addition, ultrasonic treatment, and drying methods all had no significant influence on the micromorphology and crystalline form of the lake. Ethanol addition at suitable concentration is an efficient way to improve IE of β-carotene in the lake, ultrasonic treatment had no obvious influence on the IE, and oven drying could distinctly decrease the IE in the lake. Compared to the freeze drying, the vacuum drying method supplied a compromised choice with a little impaired light stability but expected economic benefit. On the one hand, foods are stored in light-proof packaging until consumed in some cases; thus, high light stability of the foods’ color is not a key requirement, for example, the case of using the β-carotene-CaCO_3_ lake to dye dietary supplement tablets or biscuits. On the other hand, foods need to be exposed to light for display purposes in some cases; thus, high light stability is required, for example, the case of using the β-carotene-CaCO_3_ lake to dye breads or cakes. Thus, in different scenarios, production parameters of the β-carotene-CaCO_3_ lake could be adjusted to make an economical choice between color intensity (IE) and light stability.

### 3.6. Zeta Potential, Particle Size, and Surface Area

The characteristics of dried calcium carbonate, lake, β-carotene, and β-carotene suspension were compared, and the results were shown in Table 2. There were significant differences in zeta potential and diameter of the four kinds of particles (*p* < 0.05). The diameter of lake particles (8.05 μm) was significantly higher than that of calcium carbonate particles (6.85 μm) due to the adsorption of β-carotene, which had an initial diameter of 102.82 μm and a significantly reduced diameter of 0.89 μm after shearing. The results confirmed that shearing operation largely decreased the size of β-carotene suspension, which is critical for the adsorption process and formation of homogeneous lake product. In the BET results, the surface area of calcium carbonate (0.99 m^2^/g) was larger than the surface area of the lake (0.90 m^2^/g) and β-carotene (0.72 m^2^/g), which was consistent with the particle size results. The BET of β-carotene suspension was large but with much bigger deviation compared to the others, indicating that the suspended particles after shearing had a heterogeneous structure. In the process of MPs adsorption by CaCO_3_, the particle size of the lake was smaller than that of calcium carbonate and the BET of the lake was larger than that of calcium carbonate [24]. The β-carotene-CaCO_3_ colorant lake had the opposite result. The different influence of β-carotene and MPs on the size of CaCO_3_ particles could be ascribed to the distinct existing states of them in reaction solution. The results of zeta potential were consistent with those in Table 1. Calcium carbonate had the highest potential (−21.07 mV), and β-carotene was negatively charged (−28.83 mV). The results suggest that during the formation of the β-carotene-CaCO_3_ complex particles, the positively charged ions (-Ca^+^, -Ca (OH_2_)^+^) on the calcium carbonate particle surface adsorbed the negatively charged ions on the surface of β-carotene suspension, resulting in a decrease in the potential of the lake.

### 3.7. XPS and EDS Analysis

**Figure 6:**
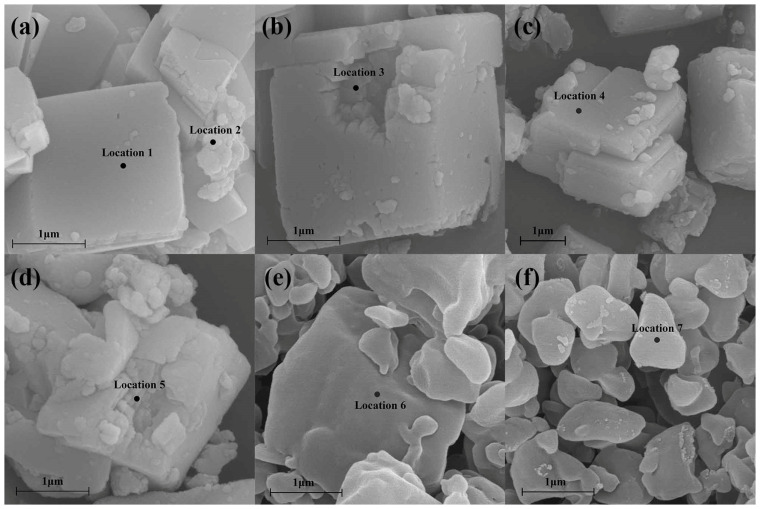
The SEM images of the β-carotene-CaCO_3_ lake (**a**,**b**), the CaCO_3_ (**c**,**d**), the β-carotene (**e**), and the β-carotene suspension (**f**), and the scanning locations (denoted by black points) for EDS analysis.

EDS is an efficient tool to evaluate elemental composition of particles [41]. The SEM images and scanning locations (denoted by black points) of the surface of lake particles (Figure 6a), internal section of lake particles (Figure 6b), surface of CaCO_3_ particles (Figure 6c), internal section of CaCO_3_ particles (Figure 6d), β-carotene particles (Figure 6e), and β-carotene suspension particles (Figure 6f) are shown in Figure 6. The atomic percentage and weight percentage obtained through EDS analysis are presented in Table 3. β-carotene (location 6) and β-carotene suspension (location 7) exhibited minimal disparity in the atomic ratios of C and O, which was expected and confirmed that shearing operation did not cause distinct change to the chemical composition of β-carotene. Location 2 was chosen for the irregular particles, which were believed to be β-carotene particles. The findings indicate that the distribution of pigment on the lake’s surface is nonuniform. In the calcium carbonate and lake samples, with the exception of location 2, all other locations exhibited atomic percentage of carbon (C) ranging from 17% to 19%, while location 2 displayed an unusually high atomic percentage of C, at approximately 33%. The results indicated that there was similar C element distribution on the surface and internal section of CaCO_3_ particles in the lake to that of pure CaCO_3_ particles, which confirmed that there was no β-carotene evenly covering the surface or on the inside of CaCO_3_ particles. The weight percentage of the samples consistently yielded results, indicating a significantly higher weight percentage of C in location 2. This finding further strengthens the conclusion drawn from the analysis based on weight percentage.

**Figure 7:**
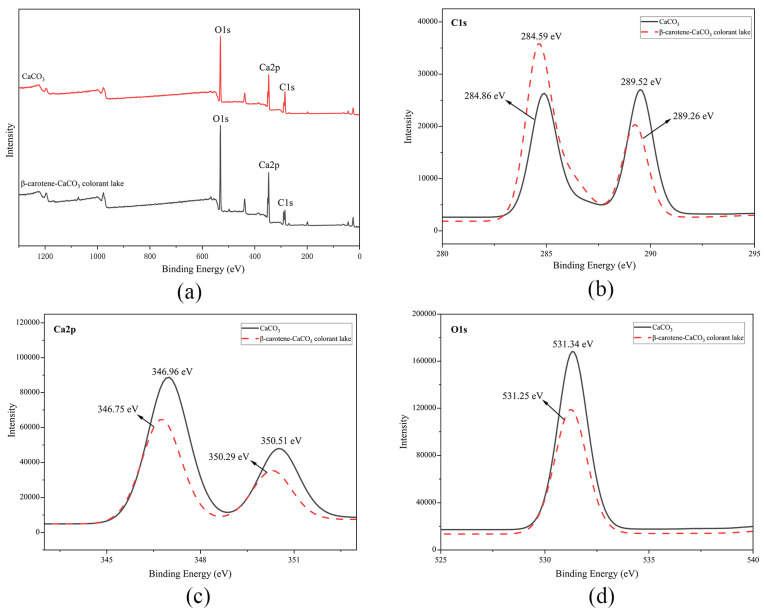
The XPS analysis (**a**) and the spectra of different elements (C1s (**b**), Ca2p (**c**), and O1s (**d**)) of the β-carotene-CaCO_3_ lake and CaCO_3._

XPS uses chemical analysis to characterize surface composition [42]. Figure 7 shows the XPS spectrum analysis results of the particle surfaces. CaCO_3_ consists of Ca, C, and O, while β-carotene solely comprises C and H. No additional measurable elements were introduced into the β-carotene-CaCO_3_ complex. Curve fitting indicated that C accounted for 47.98% in the lake and 36.75% of the CaCO_3_, indicating successful β-carotene adsorption onto the CaCO_3_ surface. The peaks corresponding to Ca, C, and O in the CaCO_3_ and color lake were analyzed for comparison. A slight shift was evident among the C, O, and Ca peaks between CaCO_3_ and the lake. This suggested a physical interaction between the β-carotene and CaCO_3_, providing further evidence for adsorption of carotene. 

Combined with the results of XPS and EDS, pigment was attached to the surface of calcium carbonate particles in the lake prepared by coprecipitation method. Calcium carbonate has little or no internal absorption of pigments.

### 3.8. TGA and DSC Analysis

**Figure 8:**
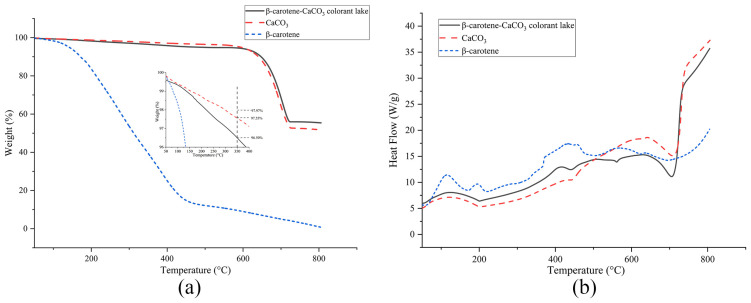
TGA (**a**) and DSC (**b**) curves of the β-carotene-CaCO_3_ lake, CaCO_3_, and β-carotene.

The TGA spectra of pigment, calcium carbonate, and the lake are presented in Figure 8a. The decomposition of the lake occurred in two distinct stages, namely, weight losses near 450 °C and 650 °C, while the decomposition of calcium carbonate only took place around 650 °C. The disparity in weight loss between the lake and calcium carbonate before 650 °C can be attributed to the influence of the pigment on calcium carbonate. The final weight loss of the lake and calcium carbonate was 44% and 41%, respectively, and the weight loss of the lake was slightly greater than that of calcium carbonate [43,44]. When the temperature was near 350 °C, the degradation of the lake was more obvious than that of CaCO_3_, indicating that β-carotene was attached to CaCO_3_ particles [45].

The DSC curve of the β-carotene (Figure 8b) exhibited significant changes, which was related to its own instability of highly unsaturated structures, while the TGA curve (Figure 7a) showed a considerable decrease in the β-carotene mass as the temperature rose from 50 °C to 425 °C. Previous studies have reported that thermal degradation of β-carotene occurs within the range of 50–150 °C [46], and the thermal cracking takes place at temperatures exceeding 300 °C [47], Consequently, multiple exothermic peaks were observed in the DSC curve. The DSC curve showed that exothermic peaks appeared in both lake and pigment at around 425 °C, while there was no CaCO_3_ peak at this temperature, which may be due to the thermal cracking of β-carotene. The adsorption of pigment by calcium carbonate was further explained.

### 3.9. FTIR Analysis

**Figure 9:**
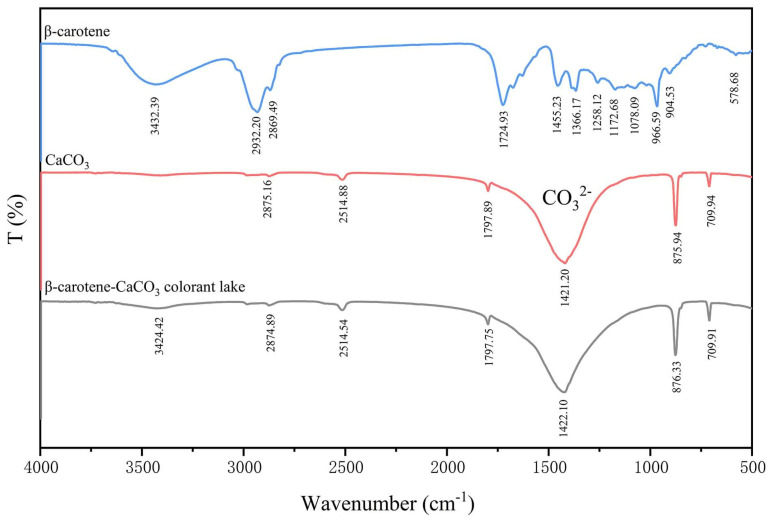
The FTIR curves of the β-carotene-CaCO_3_ lake, CaCO_3_, and β-carotene.

Figure 9 illustrates the positions and number of FTIR spectral peaks of the β-carotene, CaCO_3_, and β-carotene-CaCO_3_ colorant lakes. Table 4 shows the FTIR spectra peak assignments for CaCO_3_ and β-carotene. As shown in the figure, except for the peak at 3424.42 cm^−1^, the spectra of CaCO_3_ and β-carotene-CaCO_3_ colorant lakes have no distinct difference. The presence of peak at 3424.42 cm^−1^ in the spectrum of the lake is due to the characteristic peak in the β-carotene spectrum at similar wavenumber (3432.39 cm^−1^). No new peak is evident in the spectrum of the lake, confirming that there is no chemical bonding established between β-carotene and CaCO_3_ in the lake.

## 4. Conclusions

The preparation technology of colorant lakes can improve the stability and dyeing ability of pigments, and expand their application scope in foods. *Monascus* pigment is a water-soluble pigment and β-carotene is a fat-soluble pigment. There are big differences between the two pigments in terms of structure and function. In the literature, only colorant lake preparation techniques for water-soluble pigments have been reported, and the current manuscript presents a study on the preparation of fat-soluble pigment for the first time in the literature. The results of this study confirm that coprecipitation can be successfully used for β-carotene-CaCO_3_ lake preparation. Adsorption kinetics analysis revealed that maximum IE was attained at temperature of 40 °C and pH of 9.3. Obvious adsorption and desorption were evident during lake formation. Although ethanol, ultrasonic treatment, and drying methods influenced the lake stability and IE, they did not affect crystal morphology. The isothermal adsorption curves and EDS, XPS, TGA, DSC, and FTIR analyses demonstrated that the adsorption process was primarily governed by single-layer physical adsorption without the need for chemical reactions. Electrostatic attraction may play a predominant role in the formation of the lake. The current study demonstrates that shearing operation can significantly reduce the size of β-carotene, which is an important factor influencing the adsorption process. In future studies, more methods are expected to be investigated in order to obtain smaller sizes for β-carotene to realize better adsorption of the pigment onto CaCO_3_, such as adding food-grade reagents (ethanol, etc.) or improving the preparation method. At the same time, our team will explore the biological activity of colorant lake products and strive for the application of colorant lake products in food.

## Figures and Tables

**Table 1:** The zeta potential of different particles in reaction solution.

	pH = 8	pH = 9.3	pH = 10
β-carotene-CaCO_3_ complex particle	−26.1 ± 0.51 ^b^	−27.7 ± 0.47 ^b^	−27.3 ± 0.81 ^b^
CaCO_3_ particle	−17.1 ± 0.69 ^a^	−18.1 ± 0.54 ^a^	−17.6 ± 0.41 ^a^
β-carotene suspension	−25.8 ± 0.20 ^b^	−28.3 ± 0.26 ^b^	−27.5 ± 0.35 ^b^

Values not sharing the same superscript letters in one column are significantly different (*p* < 0.05).

**Table 2:** Basic characteristics of calcium carbonate, lake, β-carotene, and β-carotene suspension.

	Zeta Potential	Diameter	BET
	(mV)	(μm)	(m^2^/g)
CaCO_3_	−21.07 ± 0.44 ^d^	6.85 ± 0.13 ^c^	0.99 ± 0.08 ^a^
β-carotene-CaCO_3_ colorant lake	−24.37 ± 0.44 ^c^	8.05 ± 0.02 ^b^	0.90 ± 0.03 ^a^
β-carotene	−28.83 ± 0.28 ^a^	102.82 ± 5.77 ^a^	0.72 ± 0.04 ^a^
β-carotene suspension	−27.30 ± 0.62 ^b^	0.89 ± 0.01 ^d^	2.68 ± 1.29 ^b^

Values not sharing the same superscript letters in one column are significantly different (*p* < 0.05).

**Table 3:** The EDS analysis of the β-carotene-CaCO_3_ lake and CaCO_3._

		C		O		Ca		Na	
		Weight%	Atomic%	Weight%	Atomic%	Weight%	Atomic%	Weight%	Atomic%
lake	Location 1	11.25	17.37	59.64	69.16	29.11	13.47	-	-
	Location 2	22.82	32.73	52.73	56.76	24.45	10.51	-	-
	Location 3	10.74	18.58	45.01	58.47	44.25	22.95	-	-
CaCO_3_	Location 4	11.53	17.32	63.26	71.33	25.21	11.35	-	-
	Location 5	11.55	18.80	51.79	63.31	36.66	17.89	-	-
β-carotene	Location 6	72.84	78.13	27.16	21.87	-	-	-	-
β-carotene suspension	Location 7	69.81	75.99	27.51	22.48	-	-	2.68	1.52

**Table 4:** The FTIR spectral peak assignments for the CaCO_3_ and β-carotene.

	Wavenumber (cm^−1^)	Peak Assignments	References
CaCO_3_	1421.20	The asymmetric CO_3_^2−^ extension	[24,48]
	875.94	The CO_3_^2−^ plane bending vibration	
	709.94	The O-C-O group bending vibration	
β-carotene	3432.39	Stretching vibration of O-H group	[49,50,51,52,53,54]
	2869.49	Symmetric stretching vibration of C-H	
	1724.93	The C=C from the extension of the terminal cyclohexene group	
	1366.17/1455.23	The C–H from olefin	
	966.59	The C-H plane from conjugated olefin	

## Data Availability

The data presented in this study are available on request from the corresponding author. The data are not publicly available due to privacy restrictions.
